# Genome-Wide Association Studies and Haplotype-Sharing Analysis Targeting the Egg Production Traits in Shaoxing Duck

**DOI:** 10.3389/fgene.2022.828884

**Published:** 2022-03-28

**Authors:** Wenwu Xu, Zhenzhen Wang, Yuanqi Qu, Qingyi Li, Yong Tian, Li Chen, Jianhong Tang, Chengfeng Li, Guoqin Li, Junda Shen, Zhengrong Tao, Yongqing Cao, Tao Zeng, Lizhi Lu

**Affiliations:** ^1^ Institute of Animal Husbandry and Veterinary Science, Zhejiang Academy of Agricultural Sciences, Hangzhou, China; ^2^ Hubei Shendan Co., Ltd., Wuhan, China

**Keywords:** genome-wide association study, haplotype, laying duck, egg production, gene

## Abstract

Age at first egg (AFE) and egg number (EN) are economically important traits related to egg production, as they directly influence the benefits of the poultry industry, but the molecular genetic research that affects those traits in laying ducks is still sparse. Our objective was to identify the genomic regions and candidate genes associated with AFE, egg production at 43 weeks (EP43w), and egg production at 66 weeks (EP66w) in a Shaoxing duck population using genome-wide association studies (GWASs) and haplotype-sharing analysis. Single-nucleotide polymorphism (SNP)-based genetic parameter estimates showed that the heritability was 0.15, 0.20, and 0.22 for AFE, EP43w, and EP66w, respectively. Subsequently, three univariate GWASs for AFE, EP43w, and EP66w were carried out independently. Twenty-four SNPs located on chromosome 25 within a 0.01-Mb region that spans from 4.511 to 4.521 Mb were associated with AFE. There are two CIs that affect EP43w, i.e., twenty-five SNPs were in strong linkage disequilibrium region spanning from 3.186 to 3.247 Mb on chromosome 25, a region spanning from 4.442 to 4.446 Mb on chromosome 25, and two interesting genes, ACAD8 and THYN1, that may affect EP43w in laying ducks. There are also two CIs that affect EP66w, i.e., a 2.412-Mb region that spans from 127.497 to 129.910 Mb on chromosome 2 and a 0.355-Mb region that spans from 4.481 to 4.837 Mb on chromosome 29, and CA2 and GAMT may be the putative candidate genes. Our study also found some haplotypes significantly associated with these three traits based on haplotype-sharing analysis. Overall, this study was the first publication of GWAS on egg production in laying ducks, and our findings will be helpful to provide some candidate genes and haplotypes to improve egg production performance based on breeding in laying duck. Additionally, we learned from a method called bootstrap test to verify the reliability of a GWAS with small experimental samples that users can access at https://github.com/xuwenwu24/Bootstrap-test.

## Introduction

Egg production traits, including age at first egg (AFE) and egg number (EN), have always been a focus of attention in laying ducks, as they directly affect economic benefits to farmers. EN has experienced considerable genetic progress in commercial laying ducks breeds through traditional selection for several decades, reaching a level at an egg on almost every day in highly efficient laying ducks. AFE is also a very important trait for egg production, as it is a partial determination of the laying period. Nowadays, young laying ducks as early as 16 weeks of age start to produce their first egg.

So far, egg production has been greatly improved through the conventional selection strategy. However, the conventional breeding approaches are greatly influenced by the environmental effects, which unavoidably lead to inaccurate heritability estimation (Meuwissen et al., 2001; Muir 2007). We can dissect and quantify the genetic variations in egg production traits with the development of high-throughput genotyping platforms, and the genetic gain in egg production traits can be greatly increased by using a new molecular breeding strategy. Thus, identifying genetic variants affecting egg production traits is one of the primary goals in duck genetics. With the advances in technologies of molecular genetics and availability of single-nucleotide polymorphism (SNP) markers, numerous studies had been conducted to identify quantitative trait loci (QTLs) and SNPs that are associated with EN in poultry. The AnimalQTLdb website (https://www.animalgenome.org/cgi-bin/QTLdb/GG/index) reported 185 QTLs on 24 different chromosomes associated with AFE, EN, and egg production rate in chickens (Tuiskula-Haavisto et al., 2002; Sasaki et al., 2004; Schreiweis et al., 2006; Atzmon et al., 2008; Goto et al., 2011; Xu et al., 2011; Goraga et al., 2012). However, the molecular genetic research that affects egg-laying performance in laying ducks is still sparse, with only a few candidate gene studies. Some researchers have found some candidate genes, such as OIH, FSHβ, GnIH, FSHR, LRP8, VLDLR, and HSP90, to be associated with egg-laying performance in laying ducks (Xu et al., 2011; Wang et al., 2013). There are some limitations in the candidate gene study, such as the uncertainty of candidate gene selection, different genes could be heterogeneous in populations with different genetic backgrounds, the number of gene annotations of duck is small, and the function of some annotated genes is still not completely known today (Kwon and Goate 2000). Therefore, candidate gene study could not be fully utilized to analyze the molecular genetic mechanism of egg production in laying ducks.

Genome-wide association study (GWAS) has become an exceedingly effective and widely used approach in the identification of genetic variants associated with complex traits since the first application of GWAS research on age-related macular degeneration was performed successfully in 2005 by Klein et al. (Klein et al., 2005). Shaoxing duck is an excellent and high-yielding egg breed of duck, and the feeding rate reached 60% in China. After breeding, the age at the first egg of Shaoxing ducks is about 130 days, and the annual egg production can reach 300. In this study, we employed 10× whole-genome sequencing to identify the genomic regions and candidate genes associated with AFE, egg production at 43 weeks (EP43w), and egg production at 66 weeks (EP66w) in a pure line population derived from Shaoxing duck using GWASs and haplotype-sharing analyses, which could potentially accelerate the genetic improvement of egg production.

## Materials and Methods

### Ducks and Phenotypes

A total number of 166 Shaoxing ducks from Hubei Shendan Co., Ltd. (Wuhan, China) were used in our study. Blood samples were collected from brachial veins using the standard procedure in week 66. All ducks were housed in individual cages of the same condition. The AFE and weekly egg production from the onset of laying eggs to 66 weeks of age for each duck were recorded, and then the data were used to define two egg production traits, as the EN from the onset of laying eggs to 43 weeks (EP43w) and the EN from the onset of laying eggs to 66 weeks (EP66w). Animal care and use protocol was approved by the Institutional Animal Care and Use Committee of the Zhejiang Academy of Agricultural Sciences (approval number: 2021ZAASLA15), which was in accordance with the Guidelines for Experimental Animals established by the Ministry of Science and Technology (Beijing, China).

### Genome Sequencing

A standard cetyl trimethylammonium bromide (CTAB) method was used to isolate genomic DNA from blood, and agarose gel electrophoresis was used to examine the quality and quantity of DNA. After the examinations, paired-end libraries were generated for each eligible sample using standard procedures. Fragments were end-repaired, A-tailed, ligated to paired-end adaptors, and PCR amplified with 500-bp inserts for library construction. According to the manufacturer’s standard protocols, libraries were subjected to 150-bp paired-end sequencing on a HiSeq platform (Illumina, San Diego, CA, USA), to a mean sequencing depth of 10× for experimental animals. The depth ensured the accuracy of variant calling and genotyping and met the requirements for population genetic analyses.

### Variant Discovery and Genotyping

The 150-bp paired-end raw reads were aligned to the reference duck genome assembly CAU_duck1.0 with the Burrows–Wheeler alignment (BWA aln) using default parameters (Li and Durbin 2009; Huang et al., 2013). On average, 96.4% of the reads were mapped, resulting in a final average sequencing coverage of ×10 (ranging from ×8 to ×18) per individual. Mapping details of 166 resequencing samples were shown in Supplementary File S1: Supplementary Tables S1–S3. The paired reads that were mapped to the exact same position on the reference genome were marked and removed by Picard MarkDuplicates (http://broadinstitute.github.io/picard) to avoid any influence on variant detection. For GATK SNP calling, standard preprocessing (including realignment and recalibration) and calling procedures were used (DePristo et al., 2011), each sample generated its own gVCF file, and the files were merged. The output file was further filtered using VCFtools with the filter expression as QUAL < 30, QD < 2.0, MQ < 40, and FS > 60 (Danecek et al., 2011). SNPs that did not meet the following criteria were excluded: 1) a minor allele frequency >0.05; 2) maximum missing rate <0.1; and 3) only two genotypes. Identified SNPs were further classified by SnpEff based on the gene annotation of the reference genome (Cingolani et al., 2012).

### Single-Trait Genome-Wide Association Study Analysis

GEMMA (v.0.94) was employed for the single-marker association test between variants and phenotypes underlining a univariate linear mixed model (see Eq. 1) and is described in the following equation (Zhou and Stephens 2012):

$$\text{y}=\text{W}\alpha +\text{x}\beta +u+\epsilon ;\text{u }∼MVN_{n}\left(0,\lambda \tau^{-1}\text{K}\right),\;\epsilon ∼\;MVN_{n}\left(0,\lambda \tau^{-1}{\text{I}}_{n}\right)$$ (1)

$$FDR\left(P_{i}\right)=P_{i}*\frac{m}{K_{\left(P_{i}\right)}}$$ (2)

where y is the vector of phenotypic observation (AFE, EP43w, and EP66w); W is a design matrix of fixed effect, including a column of 1 s; α is a vector of fixed effects; x is a matrix of genotypes; β is the effect of SNPs; u is a vector of random effects following the multivariate normal distribution $MVN_{n}\left(0,\text{​}\text{​}\;\lambda \tau^{-1}\text{K}\right)$ , in which λ is the ratio is between ${\text{τ}}^{-1}$ and the variance of polygenetic effects, ${\text{τ}}^{-1}$ is the variance of the residual errors, and K is a kinship matrix estimated from whole-genome sequence variants; ϵ is a vector of errors following the multivariate normal distribution (see Eq. 1), and ${\text{I}}_{n}$ is an identity matrix. With high-density markers throughout the whole genome, naïve Bonferroni corrections of 0.05 divided by the number of examined SNPs to correct multiple comparisons would lead to an overly conservative threshold in our study due to the SNPs being highly correlated with each other. The empirical distribution of *p*-values of markers was used to calculate the genome-wide false discovery rate (FDR) following Storey and Benjamini (Efron and Tibshirani 1993; Yoav and Daniel 2001). The mathematic expression of the FDR is shown in Eq. 2, where m is the number of markers, $P_{i}$ is the *p*-value of the *i*th marker, and $K_{\left(P_{i}\right)}$ is the *p*-value of the *i*th marker ranked in all markers. Population stratification is one of the factors that affect the validity of a GWAS (Pearson and Manolio 2008). Quantile–quantile plots (Q-Q plots) were implemented to evaluate population stratification effects and were constructed with R software to check if stratification exists in our results.

### Post Genome-Wide Association Analysis

To detect the linkage disequilibrium (LD) of SNPs near the most significant SNPs in the GWAS results, the 3-Mb region near the top SNPs in the whole-sequence association results was used to conduct LD analysis by extracting genotypes from the data set using plink 1.07 (Pearson and Manolio 2008), and the default settings for minimum linkage between SNPs were at threshold r^2^ = 0.4. After the CIs were determined, an investigation of gene ontology (GO) for the genes within the CI was performed to determine biological processes associated with traits using the Database for Annotation, Visualization and Integrated Discovery (DAVID) (http://david.abcc.ncifcrf.gov/home.jsp) (Huang da et al., 2009).

In addition, the haplotypes in the CI were constructed by fastphase with the default setting, and an attempt to find the sharing susceptibility haplotype was made by thoroughly scanning the haplotypes of all individuals (Scheet and Stephens 2006).

### Bootstrap Test

In this study, the bootstrap test was carried out to verify the reliability of GWASs, which was a resampling technique used to estimate statistics on a population by sampling a dataset with replacement. This method can be used to estimate summary statistics such as the mean, SD, CI, or correlation coefficient, which is done by repeatedly taking small samples, calculating the statistic, and taking the average of the calculated statistics. There were two steps for the bootstrap test in this study; first, random resampling was performed 1,000 times with replacement, in which some individuals can be sampled multiple times, while some may be sampled for 0 times. Then GWASs were conducted 1,000 times to see if there were still significant signals in the susceptibility region identified in our study. Our null hypothesis of bootstrap in our study is that more than 950 out of the 1,000 GWASs did not detect significant signals in the candidate region, which indicates that the fluctuation in the data structure of our experimental population has an effect on GWASs; in other words, the significant signals obtained in the GWASs were not accidental but were caused by differences in the genomes of the experimental individuals, which were reliable (Xu et al., 2019).

## Results

### Phenotype and Genetic Parameter Statistics

Supplementary Figure S2 in Supplementary File S1 show that three phenotypes follow the normal distribution. Descriptive statistics of the AFE, EP43w, and EP66w across the whole laying period are shown in Table 1. The mean value of AFE in this population was 136.95 days, which means that Shaoxing duck started laying eggs at about 20 weeks of age. Moreover, the mean values of EP43w and EP66w were 151.27 and 268.96, respectively. Estimates of SNP-based heritability as well as phenotypic correlations between AFE, EP43w, and EP66w are displayed in Table 2. The heritability was medium for all the three phenotypes, which was 0.15, 0.20, and 0.22 for AFE, EP43w, and EP66w, respectively. Genetic correlation analyses revealed that EP43w and EP66w were positively interrelated and were negatively interrelated with AFE.

**TABLE 1:** Descriptive statistic for phenotype values.

Traits	N	Min	Max	Mean	SD
AFE	166	105.00	172.00	136.95	13.93
EP43w	166	113.00	189.00	151.27	12.59
EP66w	166	194.00	325.00	268.96	24.37

Note. AFE, age at first egg; EP43w, the egg number from onset of laying eggs to 43 weeks; EP66w, the egg number from onset of laying eggs to 66 weeks; N, number of samples; Min, the minimum of phenotype values; Max, the maximum of phenotype values.

**TABLE 2:** Estimates of SNP-based heritability (on the diagonal) and of phenotypic correlations between traits (below the diagonal).

Traits	AFE	EP43w	EP66w
AFE	0.15		
EP43w	−0.73	0.20	
EP66w	−0.34	0.59	0.22

Note. AFE, age at first egg; EP43w, the egg number from onset of laying eggs to 43 weeks; EP66w, the egg number from onset of laying eggs to 66 weeks.

### Genome-Wide Association Study

**FIGURE 1:**
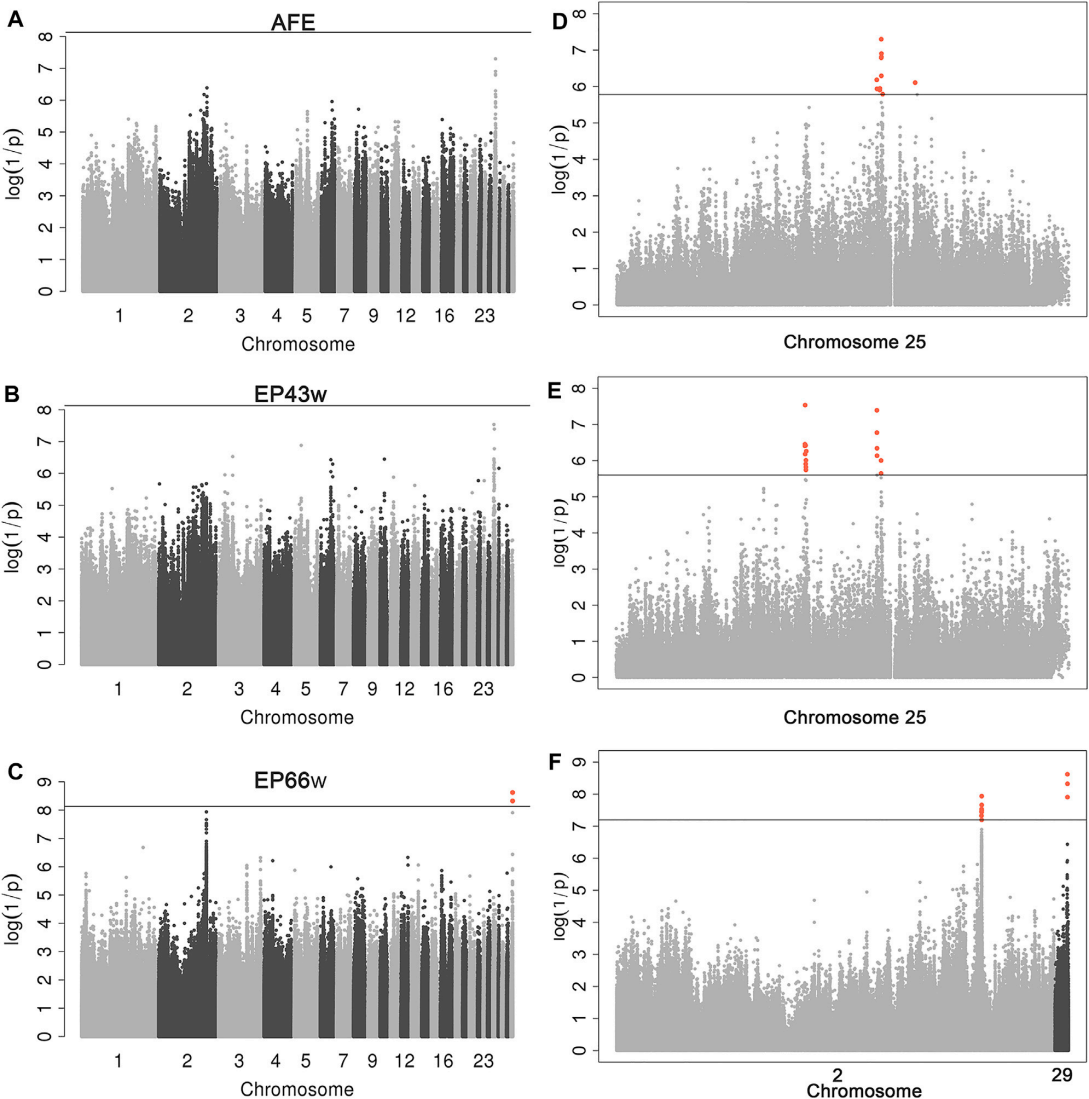
Manhattan plots derived from GWASs for AFE, EP43w, and EP66w. Each dot on this figure corresponds to a SNP within the dataset, while the *y*-axis and *x*-axis represent the negative log10 *p*-value of the SNPs and the genomic positions separated by chromosomes, respectively. Black solid lines in panels **A–C** indicate the 5% genome-wide Bonferroni-corrected threshold; the tomato puree points represent SNPs that exceeded the chromosome-wide significance threshold. Black solid lines in panels **D–F** indicate the genome-wide FDR-corrected threshold; the tomato puree points represent SNPs that exceeded this threshold. GWASs, genome-wide association studies; AFE, age at first egg; EP43w, egg production at 43 weeks; EP66w, egg production at 66 weeks; SNP, single-nucleotide polymorphism; FDR, false discovery rate.

**FIGURE 2:**
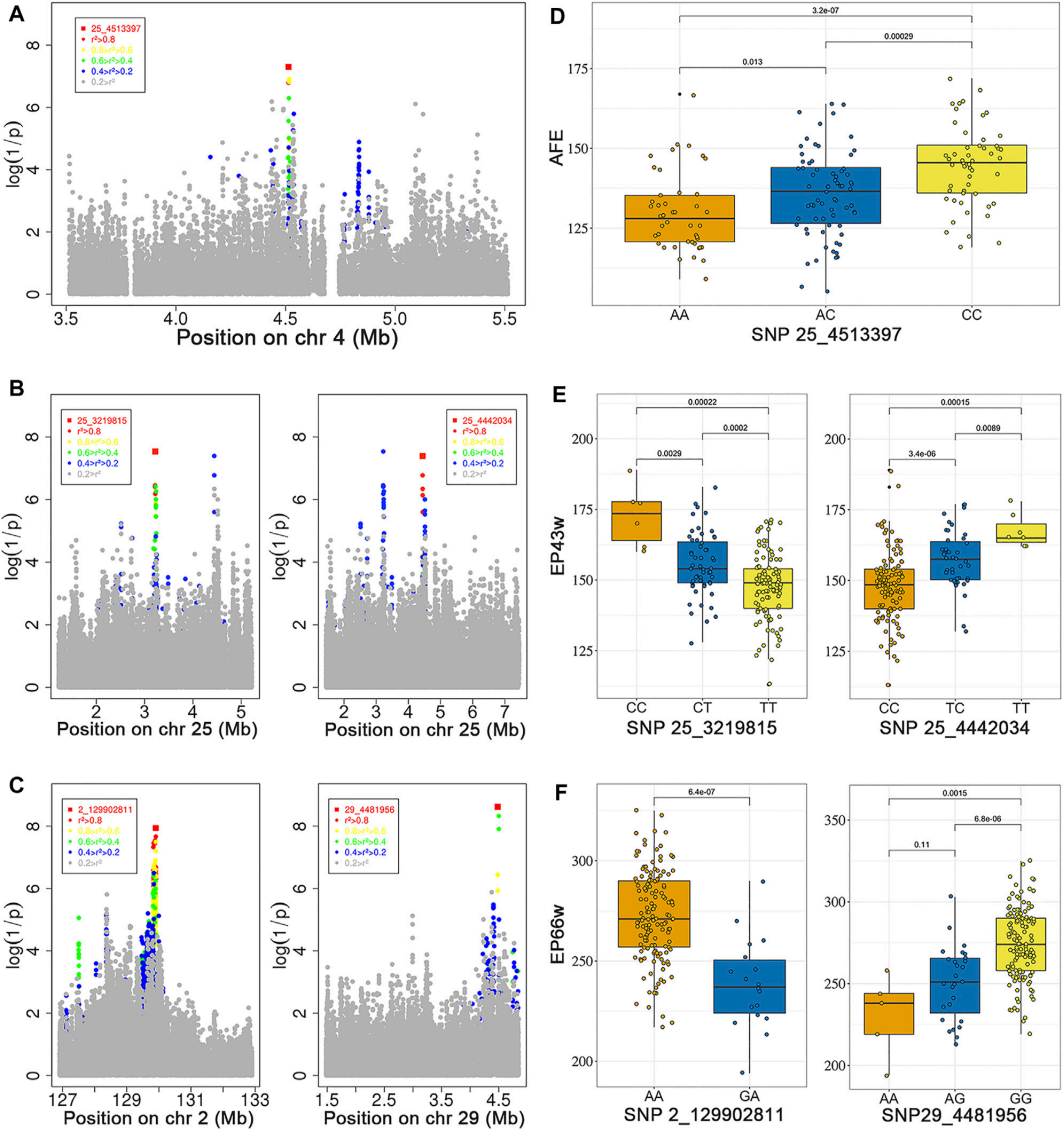
Regional plots for the strongly associated region in the GWAS for AFE **(A)**, EP43w **(B),** and EP66w **(C)**. The horizontal coordinates indicate the strongly associated region, and the vertical coordinates indicate the *p*-values; the color of each locus indicates the LD status in the most significantly associated locus. Distribution of phenotypic values for the three SNP genotypes most associated with AFE **(D)**, EP43w **(E),** and EP66w **(F)**. GWAS, genome-wide association study; AFE, age at first egg; EP43w, egg production at 43 weeks; EP66w, egg production at 66 weeks; LD, linkage disequilibrium; SNP, single-nucleotide polymorphism.

After quality control, a total of 6,746,746 SNPs and 166 individuals were retained for further analyses. Association tests for AFE, EP43w, and EP66w were performed using a univariate linear model, and the threshold obtained by the naïve Bonferroni was 7.41E−09. The result showed that there was no SNP in the AFE and EP43w that surpassed this threshold, except for two SNPs on chromosome 29 that surpassed this threshold for EP66w (Figures 1A–C). It was easy to detect that the most significant sites appeared on chromosome 25 for AFE and EP43w, so we performed FDR correction on the *p*-values of those sites on chromosome 25, and all significantly associated loci that surpassed the FDR corrective threshold are shown in Table 3. In detail, we identified 12 SNPs that surpassed the FDR corrective genome-wide significance level for AFE (Figure 1D), and the most significantly associated SNP 25_4513397 (P_wald = 5.01E−08, Qvalue = 3.24E−03) was located at 4,513,397 bp within a 0.68-Mb region (4.44–5.12 Mb) on chromosome 25 (Figure 2D). We identified a total of 17 SNPs that surpassed the FDR corrective genome-wide significance level for EP43w (Figure 1E), and the most significantly associated SNP 25_3219815 (*p*-value = 2.91E−08, Qvalue = 1.881E−03) was located at 3,219,815 bp on chromosome 25 (Figure 2E). In addition, there was another QTL (4,442,034–4,513,397 bp) on chromosome 25 also associated with EP43w, and the most significantly associated SNP was 25_4442034 (P_wald = 4.05E−08, Qvalue = 1.309E−03) (Figure 2E). For the EP66w trait, we also identified 9 and 3 SNPs on chromosome 2 and chromosome 29 significantly associated with EP66w, respectively; the most associated SNP 2_129902811 (P_wald = 1.15E−08, Qvalue = 4.075E−03) and SNP 29_4481956 (P_wald = 2.39E−09, Qvalue = 2.535E−03) were located at 129,902,811 bp on chromosome 2 and 4,481,956 bp on chromosome 25, respectively (Figure 2F). In addition, to validate the possibility of spurious SNPs caused by population stratification, the Q-Q plots for these GWASs were explored (Supplementary Figure S1). The average inflation factors (*λ*) of the GWASs were 1.01, 1.02, and 1.01 in the three traits, indicating that population structures were properly corrected.

**TABLE 3:** Description of the significant SNPs associated with AFE, EP43w, and EP66w.

EP43w				AFE				EP66w			
Chr	Position	P_wald	Qvalue	Chr	Position	P_wald	Qvalue	Chr	Position	P_wald	Qvalue
25	3,219,815	2.91E−08	1.881E−03	25	4,513,397	5.01E−08	3.243E−03	29	4,481,956	2.39E−09	2.535E−03
25	4,442,034	4.05E−08	1.309E−03	25	4,516,366	1.24E−07	4.010E−03	29	4,500,604	4.75E−09	2.517E−03
25	4,442,632	1.68E−07	3.621E−03	25	4,515,630	1.53E−07	3.302E−03	2	129,902,811	1.15E−08	4.075E−03
25	3,216,505	3.52E−07	5.684E−03	25	4,513,382	1.62E−07	2.613E−03	29	4,500,595	1.24E−08	3.280E−03
25	3,227,771	3.80E−07	4.909E−03	25	4,514,932	5.06E−07	6.547E−03	2	129,903,599	2.17E−08	4.600E−03
25	3,216,680	3.91E−07	4.217E−03	25	4,436,853	6.52E−07	7.034E−03	2	129,877,347	2.94E−08	5.184E−03
25	4,443,950	4.56E−07	4.209E−03	25	5,092,232	7.77E−07	7.184E−03	2	129,903,026	3.22E−08	4.869E−03
25	3,238,808	5.49E−07	4.436E−03	25	4,490,115	1.12E−06	9.051E−03	2	129,836,874	3.51E−08	4.645E−03
25	3,220,324	6.57E−07	4.721E−03	25	4,441,061	1.15E−06	8.271E−03	2	129,877,317	3.61E−08	4.255E−03
25	4,444,559	7.30E−07	4.724E−03	25	4,489,078	1.25E−06	8.065E−03	2	129,877,399	3.61E−08	3.829E−03
25	3,233,091	9.80E−07	5.764E−03	25	4,537,557	1.61E−06	9.480E−03	2	129,826,588	4.70E−08	4.530E−03
25	4,513,397	9.92E−07	5.345E−03	25	5,126,926	1.65E−06	8.903E−03	2	129,903,609	6.34E−08	5.598E−03
25	3,229,361	1.25E−06	6.197E−03								
25	3,232,968	1.52E−06	7.017E−03								
25	3,232,878	1.81E−06	7.798E−03								
25	4,513,382	2.26E−06	9.150E−03								
25	4,444,987	2.51E−06	9.550E−03								

Note. Chr, chromosome number; position, base positions on the chromosome; P_wald, *p*-value from the wald test; Qvalue, *p*-value corrected by FDR; SNPs, single-nucleotide polymorphisms; AFE, age at first egg; EP43w, the egg number from onset of laying eggs to 43 weeks; EP66w, the egg number from onset of laying eggs to 66 weeks; FDR, false discovery rate.

### Post Genome-Wide Association Analysis

Through LD (Atzmon et al.) analysis, for AFE trait, we identified 23 SNPs that have strong LD status in the most significantly associated SNP 25_4513397 (Figure 2A, Supplementary Table S4), which were located within a 0.01-Mb region that spans from 4.511 to 4.521 Mb on chromosome 25. The candidate genes within the 0.01-Mb region included GRIK4 and ARHGEF12. For EP43w, we identified 25 SNPs that have strong LD status in the most significantly associated SNP 25_3219815 (Figure 2B, Supplementary Table S4), which were located within a 0.06-Mb region that spans from 3.186 to 3.247 Mb on chromosome 25. The candidate genes within the 0.06-Mb region involved five genes, including B3GAT1, VPS26B, ACAD8, THYN1, and NCAPD3. On chromosome 25, we also identified 8 SNPs that have strong LD status in the significantly associated SNP 25_4442034 (Figure 2B, Supplementary Table S4), which were located within a 0.02-Mb region that spans from 4.442 to 4.446 Mb. For the EP66w trait, we identified 318 SNPs that have strong LD status in the most significantly associated SNP 2_129902811 (Figure 2C, Supplementary Table S4), which were located within a 2.412-Mb region that spans from 127.497 to 129.910 Mb on chromosome 2. The candidate genes of EP66w within the 2.412 Mb involved six genes, including RALYL, LRRCC1, E2F5, RBIS, CA13, and CA2. We also identified 17 SNPs that have strong LD status in the most significantly associated SNP 29_4481956 (Figure 2C, Supplementary Table S4), which were located within a 0.355-Mb region that spans from 4.481 to 4.837 Mb on chromosome 29. The candidate genes within the 0.355-Mb region involved 13 genes, including DAZAP1, GAMT, NDUFS7, CIRBP, FAM174C, MIDN, STK11, SBNO2, POLR2E, ARHGAP45, GRIN3B, TMEM259, and WDR18. Overall, we identified a total of 26 candidate genes associated with the AFE, EP43w, and EP66w traits. Next, these genes were used to perform GO based on biological process analysis in DAVID (available at http://david.abcc.ncifcrf.gov/home.jsp), nine significant GO terms were identified (Supplementary Figure S3, Supplementary Table S5), and most genes are enriched in cytoplasm term and cytosol term.

### Haplotype-Sharing Analysis

**FIGURE 3:**
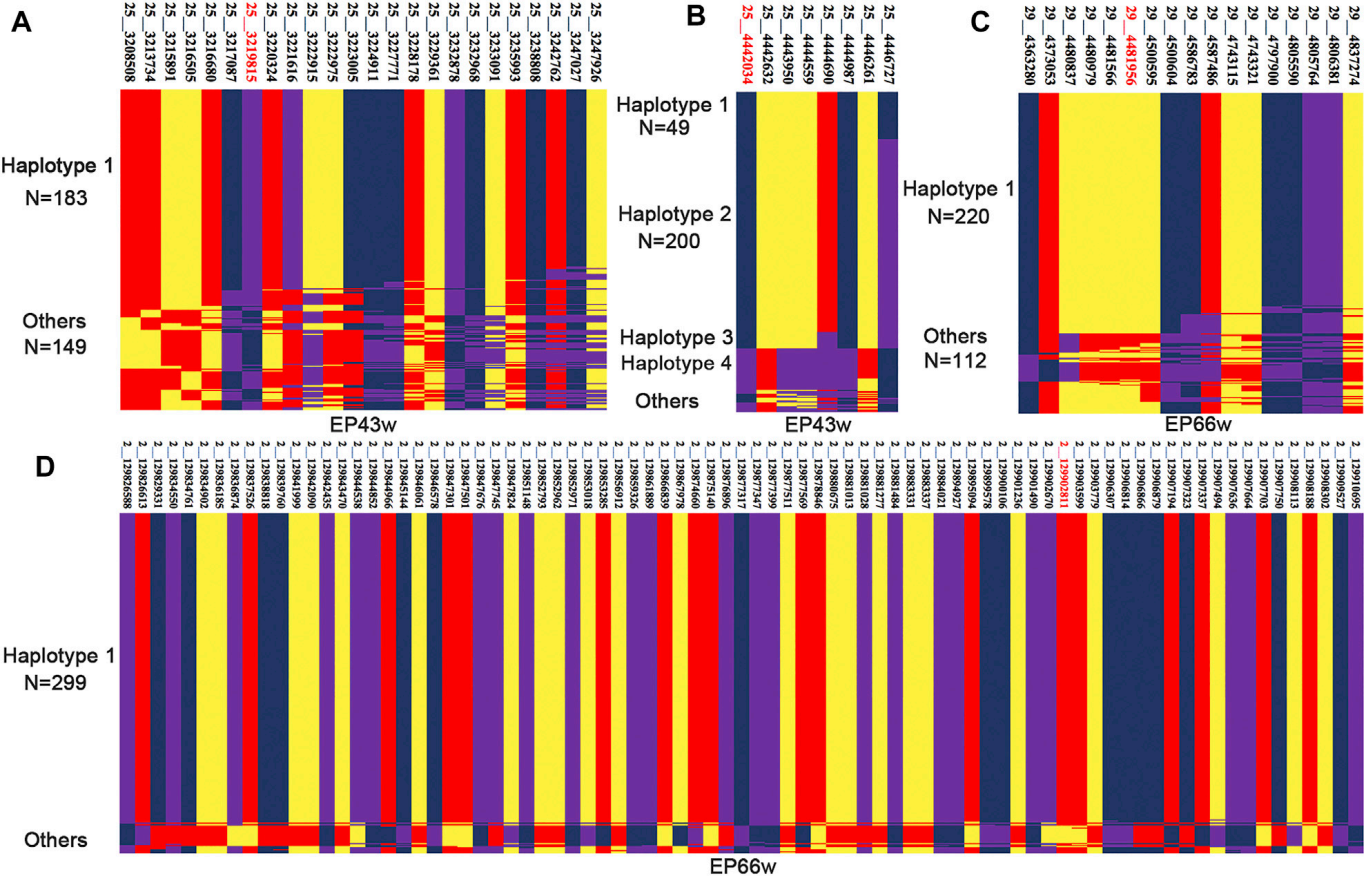
The haplotype sharing within the CI for EP43w and EP66w. EP43w, egg production at 43 weeks; EP66w, egg production at 66 weeks.

Through LD analysis, we obtained some corresponding CIs for AFE, EP43w, and EP66w, and then we performed a haplotype-sharing analysis of these intervals. The results are shown in Figure 3A and Supplementary Table S6 in Supplementary File S2. We found that 184 sequences shared a type of haplotype for EP43w, defined as haplotype 1; the mean value of haplotype 1 with a corresponding phenotype was 148.86; the other haplotypes consisted of the remaining 149 sequences without any regularity, so we defined them as chaotic haplotypes, and the corresponding mean of those phenotypes was 154.22. Next, we carried out a *t*-test with haplotype 1 and chaotic haplotype (*p*-value = 0.0001), which indicated that haplotype 1 has a significant effect on EP43w. In addition, we also found four haplotypes in the CI (4,442,034 to 4,446,727 bp) that were related to EP43w (Figure 3B and Supplementary Table S7 in Supplementary File S2) and named them haplotype 1, haplotype 2, haplotype 3, and haplotype 4, respectively, with the mean of 147.2, 149.9, 148.5, and 159.5 for the corresponding phenotypes. As there is no difference between haplotypes 1, 2, and 3, we merged those three haplotypes and did a *t*-test with haplotype 4, resulting in a *p*-value of 4.19E−05, which indicated that of the haplotypes, haplotype 4 has a significant effect of increasing EP43w. As the results show in Figure 3C and Supplementary Table S8 in Supplementary File S2, we found that there were 220 sequences located on chromosome 29 that shared a type of haplotype for EP66w, defined as haplotype 1. The mean value of haplotype 1 with a corresponding phenotype was 272.89. The other haplotypes consisted of the remaining 112 sequences also without any regularity and are defined as chaotic haplotypes, with a corresponding mean of phenotypes of 260.4. The result of the *t*-test with haplotype 1 and chaotic haplotype (*p*-value = 2.8E−05) is indicative that haplotype 1 has a significant effect of increasing EP66w. For EP66w (Figure 3D and Supplementary Table S9 in Supplementary File S2), another CI that spans from 127.497 to 129.910 Mb on chromosome 2 contained 318 loci, and we selected the loci with LD > 0.8 for haplotype-sharing analysis. The result showed that 299 sequences shared a type of haplotype (haplotype 1), the mean value of haplotype 1 with a corresponding phenotype was 271.94, and the other haplotypes consisting of the remaining 33 sequences were also defined as chaotic haplotypes, which correspond to the mean of those phenotypes at 241.87. Then we carried out a *t*-test with haplotype 1 and chaotic haplotype (*p*-value = 1.05E−09), which indicated that haplotype 1 has a significant effect on EP66w.

### Bootstrap Test

Although these studies revealed some crucial discoveries, there were some limitations, such as the relatively small number of samples in our experimental population. Therefore, we herein carried out a bootstrap test to verify the reliability of GWASs in our study. For trait EP43w, there are 985 of the 1,000 GWASs that did not detect significant signals (P_wald < 1.81E−06) in the interval from 3,216,505 to 3,238,808 bp on chromosome 25, and there are 931 of the 1,000 GWASs that did not detect significant signals in the interval from 4,442,034 to 4,513,397 bp on chromosome 25. For trait EP66w, there are 987 of the 1,000 GWASs that did not detect significant signals (P_wald < 6.34E−08) in the interval from 129,826,588 to 129,903,609 bp on chromosome 2, and there are 992 of the 1,000 GWASs that did not detect significant signals (P_wald < 1.24E−08) in the interval from 4,481,956 to 4500595 bp on chromosome 29. These results indicated that the fluctuation in the data structure of our experimental population has an effect on GWASs (FDR < 0.05); in other words, the significant signals obtained in our GWAS were not accidental but were caused by differences in the genomes of the experimental individuals, which were reliable.

## Discussion

Egg production is an important economic trait. So far, many studies have focused on the genetic determinants of AFE, EP43w, and EP66w in chicken and have reported some candidate QTLs and genes (Liu et al., 2011; Goraga et al., 2012; Wolc et al., 2014; Yuan et al., 2015; Kudinov et al., 2019; Liu et al., 2019). However, the molecular genetic research that affects egg-laying performance in laying ducks is still limited, with only a few candidate gene studies. GWAS has become a powerful approach for genetic dissection of trait loci along with the completion of genome sequencing and the development of a high-density SNP array. In our study, we performed a GWAS for AFE, EP43w, and EP66w using a univariate linear mixed model. This is the first GWAS that used the whole-genome sequencing in a Shaoxing pure line population across the whole laying period.

Genetic parameter estimates show that AFE, EP43w, and EP66w are medium heritable traits, which approximately coincided with the report by Chen et al. (Chen and Tan 1996). Our research is the first report of heritability estimates of egg production in laying ducks using the whole-genome sequencing, which can provide some reference for subsequent studies on egg production in laying ducks.

We conducted a GWAS in Shaoxing duck population and provided strong evidence of the association of SNPs with 3 traits of egg production. There is an LD between the marker SNP and the causative variation within or near genes, as most SNPs found at genome-wide significance level in our study are within the known genes. Identifications of these loci may provide new insights into the genetic basics of egg production traits, though the characteristics and functions of these genes have not been studied in depth.

Number of eggs and AFE are two important production traits in laying ducks, and producing laying duck with earlier sexual maturity and a higher rate has always been the goal of laying duck breeding. Our study indicated that these reproductive traits are sex-limited and have low-to-moderate heritability, indicating that they can be genetically improved by marker-assisted selection and genomic selection. In this study, we found two candidate genes that affect AFE, including GRIK4 and ARHGEF12, and we found five candidate genes that affect EP43w, including B3GAT1, VPS26B, ACAD8, THYN1, and NCAPD3. ACADs are a family of mitochondrial flavoenzymes that catalyze the dehydrogenation steps of the α- and β-oxidation processes, which are related to fatty acid β-oxidation. Lv et al. have found that dietary genistein supplementation in feed inhibited fatty acid synthesis and enhanced β-oxidation in the livers of layers with fatty liver syndrome through the PPAR–ACAD pathways, thereby alleviating fat deposition and lipid metabolism disorder, resulting in significant improvement in the laying rate poultry (Lv et al., 2018). Yuan et al. found THYN1 was associated with immune and cytokines, which played essential modulatory roles in the regulation of ovarian function (Onagbesan et al., 2009; Yuan et al., 2015). We found six genes located in CHROMOSOME 2 that affect EP66w, including RALYL, LRRCC1, E2F5, RBIS, CA13, and CA2. Carbonic anhydrase II (CA2) is a widespread zinc metalloenzyme from the carbonic anhydrase family and is essential for osteoclast activity, hydration of carbon dioxide, and pH balance (Roth et al., 1992; Geers and Gros, 2000). Nys and de Laage reported that the level of carbonic anhydrase is lower in the uterus and duodenum of hens laying soft-shelled eggs (Nys and de Laage, 1984). Some studies have proposed that disrupted carbonic anhydrase expression and distribution are involved in the mechanism of estrogen-induced eggshell thinning (Holm et al., 2001; Berg et al., 2004). Dunn et al. reported that CA2 gene polymorphism is associated with chicken egg shape (Dunn et al., 2009). Especially, Chang et al. found that CA2 is one of the differentially expressed transcripts in the duck isthmus epithelium during the egg formation period, and they confirmed that some SNPs in the 3′-UTR of the CA2 gene in Tsaiya ducks are associated with egg reproduction traits (Chang et al., 2013). We found 13 genes located in CHROMOSOME 29 that affect EP66w, including DAZAP1, GAMT, NDUFS7, CIRBP, FAM174C, MIDN, STK11, SBNO2, POLR2E, ARHGAP45, GRIN3B, TMEM259, and WDR18. Guanidinoacetate *N*-methyltransferase (GAMT) has been shown to be associated with the reproductive system and development, which implies that GAMT may be a candidate gene underlying egg production traits (Singh et al., 2019). In addition, our study also found some haplotypes that were significantly associated with these three traits, which can be helpful to improve egg production performance in laying duck based on breeding.

The relatively small number of samples in our experimental population is a limitation of this study. Therefore, we refer to a method called the bootstrap test to verify the reliability of GWASs in this study. The result showed that significant signals obtained in our GWASs were not accidental and were reliable. We have uploaded this method to the GitHub website, and users can access this method at https://github.com/xuwenwu24/Bootstrap-test.

## Conclusion

In summary, this study demonstrates that AFE, EP43w, and EP66w have medium heritability, and there were strong correlations between them. We have located some significant confidence regions for those traits, and some genes, such as GRIK4 ARHGEF12, ACAD8, THYN1, CA2, and GAMT, may be the putative candidate genes underlying this interval based on its biochemical and physiological functions. In addition, our study also found some haplotypes that were significantly associated with these three traits. Post-study can identify causal mutations by enriching markers within the identified intervals and functional studies on related genes.

## Data Availability

The datasets presented in this study can be found in online repositories. The names of the repository/repositories and accession number(s) can be found below: https://ngdc.cncb.ac.cn, PRJCA005720.
